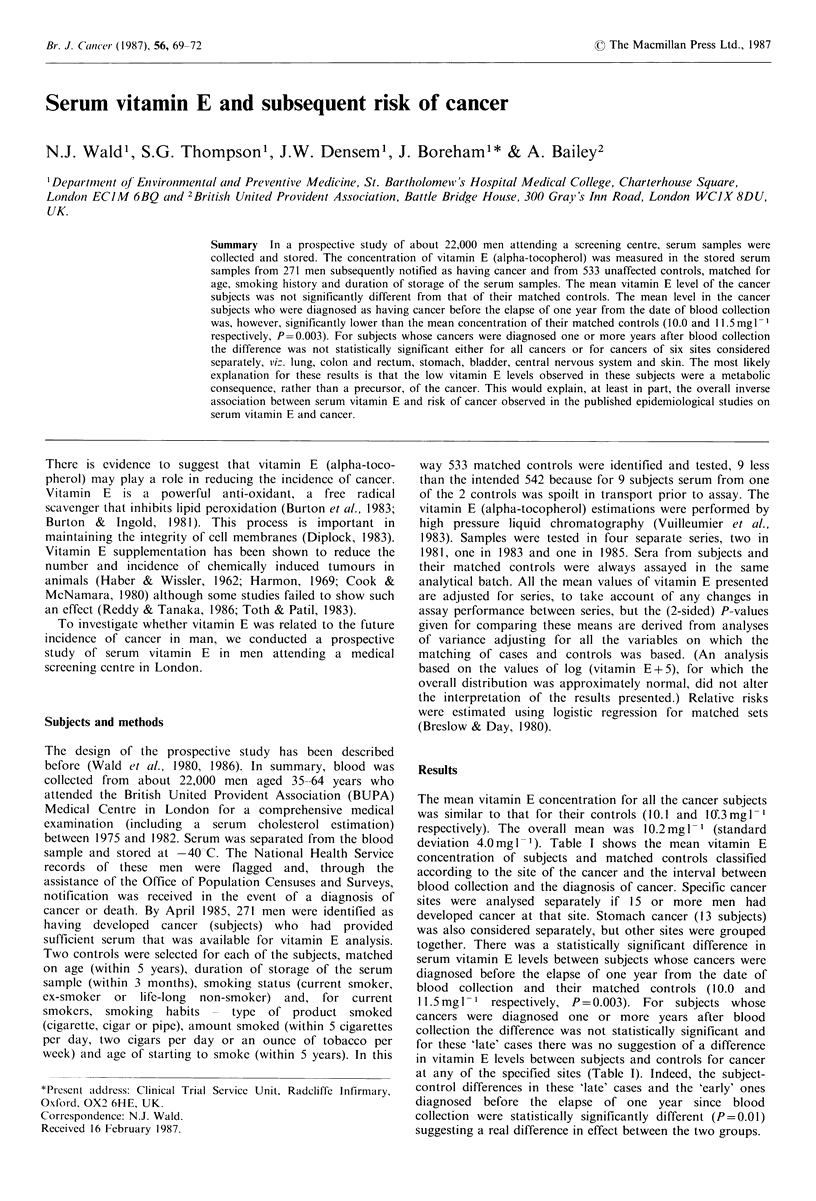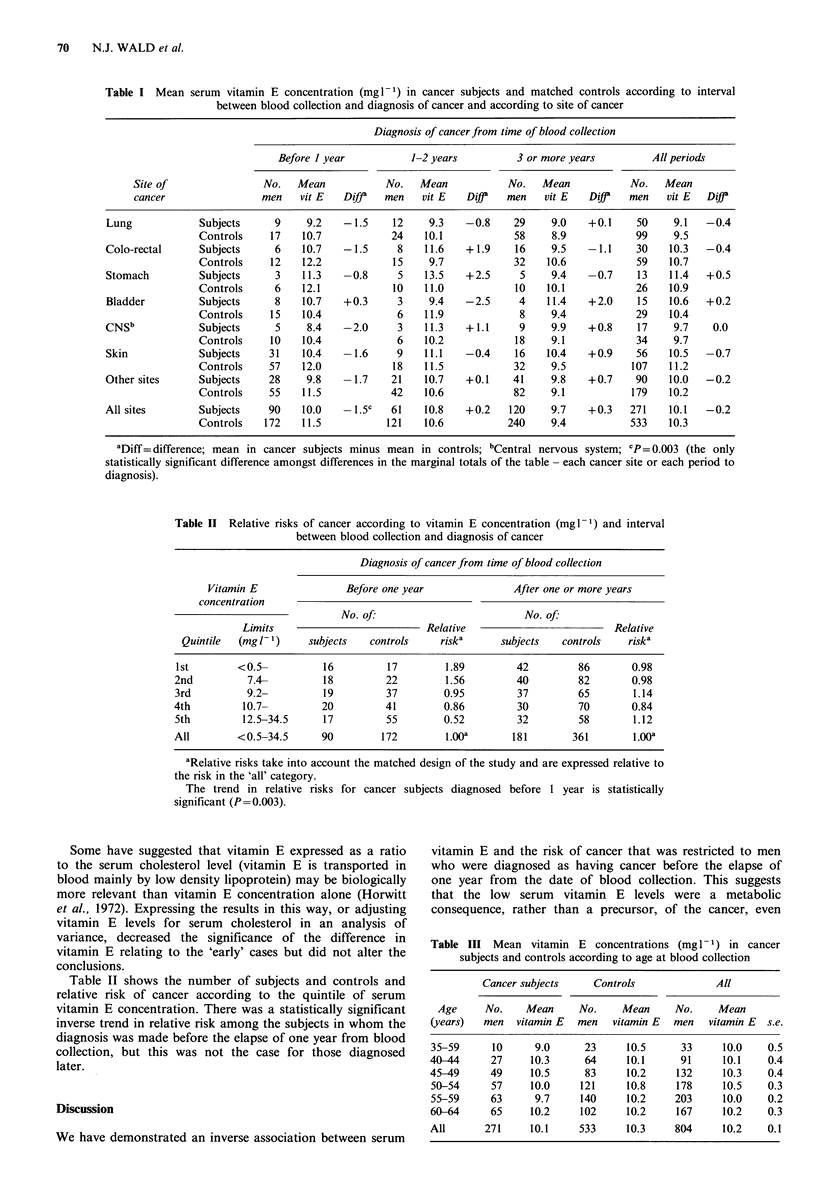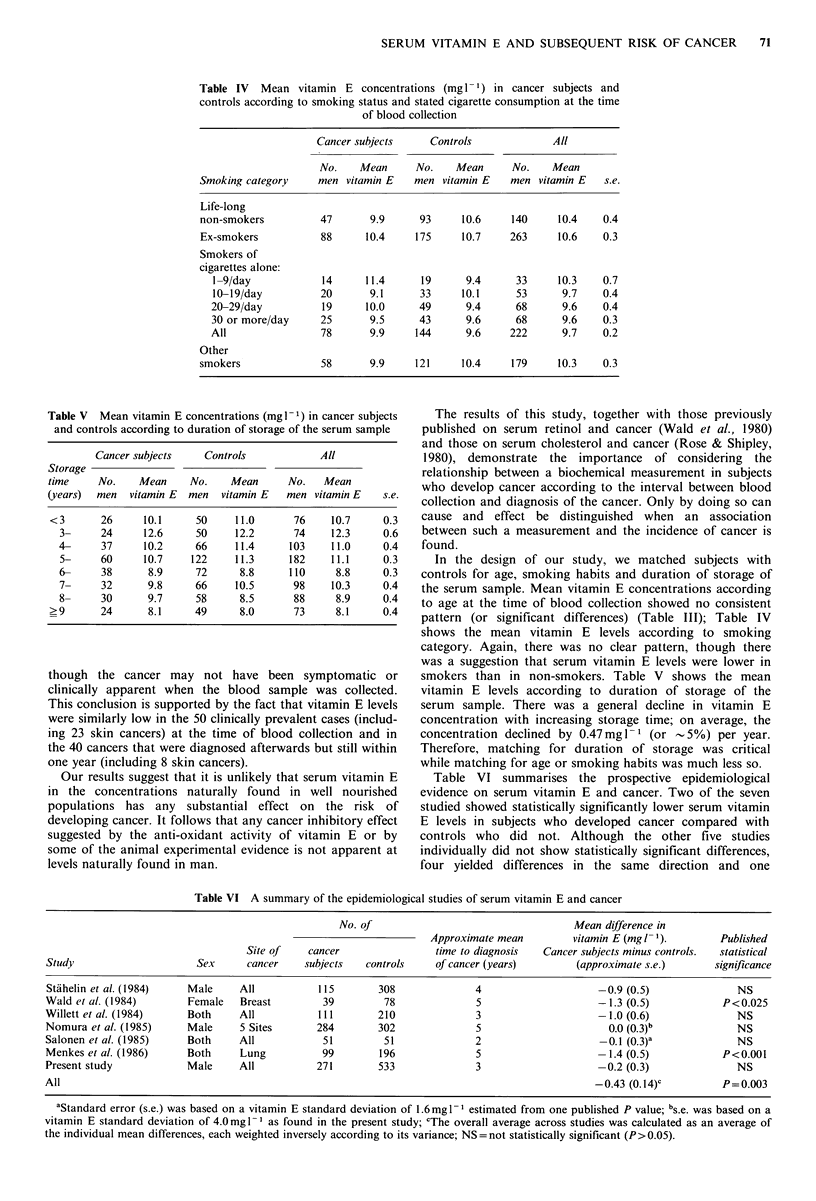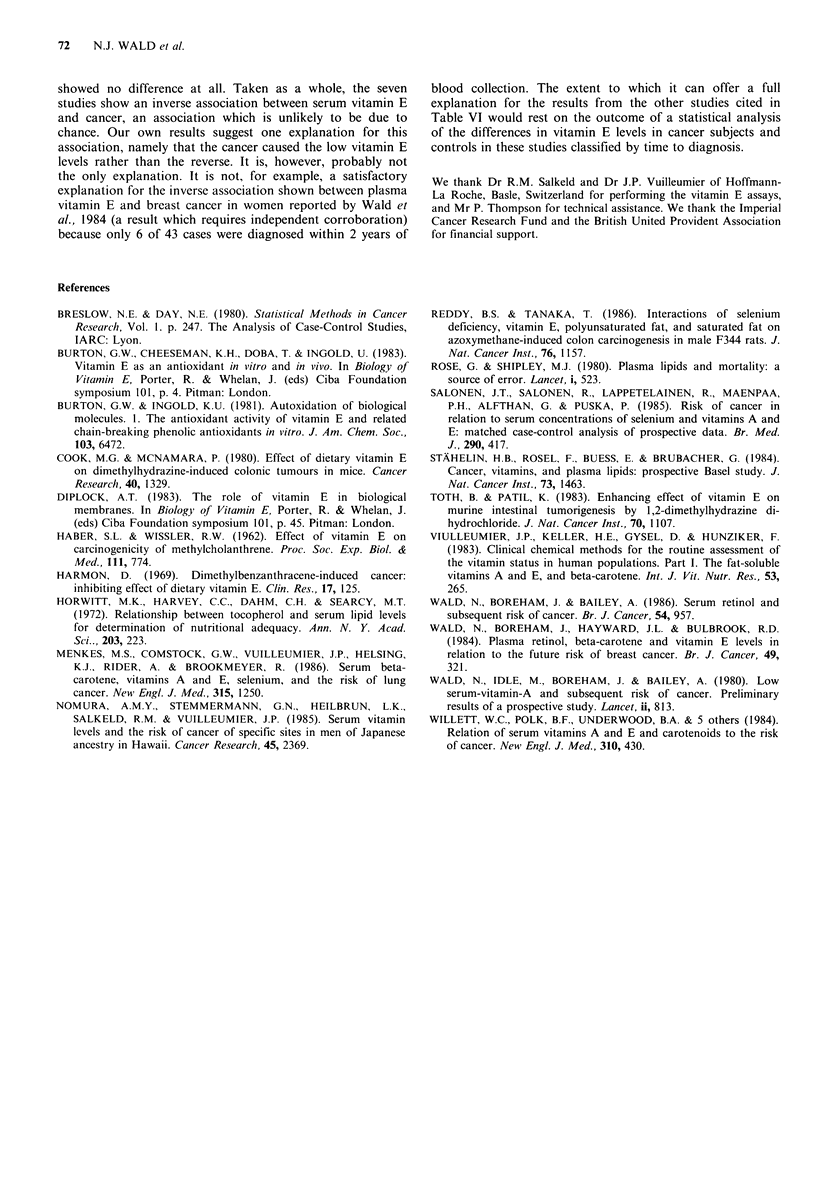# Serum vitamin E and subsequent risk of cancer.

**DOI:** 10.1038/bjc.1987.156

**Published:** 1987-07

**Authors:** N. J. Wald, S. G. Thompson, J. W. Densem, J. Boreham, A. Bailey

## Abstract

In a prospective study of about 22,000 men attending a screening centre, serum samples were collected and stored. The concentration of vitamin E (alpha-tocopherol) was measured in the stored serum samples from 271 men subsequently notified as having cancer and from 533 unaffected controls, matched for age, smoking history and duration of storage of the serum samples. The mean vitamin E level of the cancer subjects was not significantly different from that of their matched controls. The mean level in the cancer subjects who were diagnosed as having cancer before the elapse of one year from the date of blood collection was, however, significantly lower than the mean concentration of their matched controls (10.0 and 11.5 mgl-1 respectively, P = 0.003). For subjects whose cancers were diagnosed one or more years after blood collection the difference was not statistically significant either for all cancers or for cancers of six sites considered separately, viz. lung, colon and rectum, stomach, bladder, central nervous system and skin. The most likely explanation for these results is that the low vitamin E levels observed in these subjects were a metabolic consequence, rather than a precursor, of the cancer. This would explain, at least in part, the overall inverse association between serum vitamin E and risk of cancer observed in the published epidemiological studies on serum vitamin E and cancer.


Br. J. Cancer (1987), 56, 69–72 © The Macmillan Press Ltd., 1987

### Serum vitamin E and subsequent risk of cancer

N.J. Wald[1], S.G. Thompson[1], J.W. Densem[1], J. Boreham[1]* & A. Bailey[2]

[1]*Department of Environmental and Preventive Medicine, St. Bartholomew's Hospital Medical College, Charterhouse Square, London EC1M 6BQ and* [2]*British United Provident Association, Battle Bridge House, 300 Gray's Inn Road, London WC1X 8DU, UK.*

**Summary** In a prospective study of about 22,000 men attending a screening centre, serum samples were collected and stored. The concentration of vitamin E (alpha-tocopherol) was measured in the stored serum samples from 271 men subsequently notified as having cancer and from 533 unaffected controls, matched for age, smoking history and duration of storage of the serum samples. The mean vitamin E level of the cancer subjects was not significantly different from that of their matched controls. The mean level in the cancer subjects who were diagnosed as having cancer before the elapse of one year from the date of blood collection was, however, significantly lower than the mean concentration of their matched controls (10.0 and 11.5 mg l$^{-1}$ respectively, $P=0.003$). For subjects whose cancers were diagnosed one or more years after blood collection the difference was not statistically significant either for all cancers or for cancers of six sites considered separately, *viz*. lung, colon and rectum, stomach, bladder, central nervous system and skin. The most likely explanation for these results is that the low vitamin E levels observed in these subjects were a metabolic consequence, rather than a precursor, of the cancer. This would explain, at least in part, the overall inverse association between serum vitamin E and risk of cancer observed in the published epidemiological studies on serum vitamin E and cancer.

There is evidence to suggest that vitamin E (alpha-tocopherol) may play a role in reducing the incidence of cancer. Vitamin E is a powerful anti-oxidant, a free radical scavenger that inhibits lipid peroxidation (Burton *et al.*, 1983; Burton & Ingold, 1981). This process is important in maintaining the integrity of cell membranes (Diplock, 1983). Vitamin E supplementation has been shown to reduce the number and incidence of chemically induced tumours in animals (Haber & Wissler, 1962; Harmon, 1969; Cook & McNamara, 1980) although some studies failed to show such an effect (Reddy & Tanaka, 1986; Toth & Patil, 1983).

To investigate whether vitamin E was related to the future incidence of cancer in man, we conducted a prospective study of serum vitamin E in men attending a medical screening centre in London.

### Subjects and methods

The design of the prospective study has been described before (Wald *et al.*, 1980, 1986). In summary, blood was collected from about 22,000 men aged 35–64 years who attended the British United Provident Association (BUPA) Medical Centre in London for a comprehensive medical examination (including a serum cholesterol estimation) between 1975 and 1982. Serum was separated from the blood sample and stored at −40°C. The National Health Service records of these men were flagged and, through the assistance of the Office of Population Censuses and Surveys, notification was received in the event of a diagnosis of cancer or death. By April 1985, 271 men were identified as having developed cancer (subjects) who had provided sufficient serum that was available for vitamin E analysis. Two controls were selected for each of the subjects, matched on age (within 5 years), duration of storage of the serum sample (within 3 months), smoking status (current smoker, ex-smoker or life-long non-smoker) and, for current smokers, smoking habits – type of product smoked (cigarette, cigar or pipe), amount smoked (within 5 cigarettes per day, two cigars per day or an ounce of tobacco per week) and age of starting to smoke (within 5 years). In this way 533 matched controls were identified and tested, 9 less than the intended 542 because for 9 subjects serum from one of the 2 controls was spoilt in transport prior to assay. The vitamin E (alpha-tocopherol) estimations were performed by high pressure liquid chromatography (Vuilleumier *et al.*, 1983). Samples were tested in four separate series, two in 1981, one in 1983 and one in 1985. Sera from subjects and their matched controls were always assayed in the same analytical batch. All the mean values of vitamin E presented are adjusted for series, to take account of any changes in assay performance between series, but the (2-sided) $P$-values given for comparing these means are derived from analyses of variance adjusting for all the variables on which the matching of cases and controls was based. (An analysis based on the values of log (vitamin E+5), for which the overall distribution was approximately normal, did not alter the interpretation of the results presented.) Relative risks were estimated using logistic regression for matched sets (Breslow & Day, 1980).

*Present address: Clinical Trial Service Unit, Radcliffe Infirmary, Oxford, OX2 6HE, UK.

Correspondence: N.J. Wald.

Received 16 February 1987.

### Results

The mean vitamin E concentration for all the cancer subjects was similar to that for their controls (10.1 and 10.3 mg l$^{-1}$ respectively). The overall mean was 10.2 mg l$^{-1}$ (standard deviation 4.0 mg l$^{-1}$). Table I shows the mean vitamin E concentration of subjects and matched controls classified according to the site of the cancer and the interval between blood collection and the diagnosis of cancer. Specific cancer sites were analysed separately if 15 or more men had developed cancer at that site. Stomach cancer (13 subjects) was also considered separately, but other sites were grouped together. There was a statistically significant difference in serum vitamin E levels between subjects whose cancers were diagnosed before the elapse of one year from the date of blood collection and their matched controls (10.0 and 11.5 mg l$^{-1}$ respectively, $P=0.003$). For subjects whose cancers were diagnosed one or more years after blood collection the difference was not statistically significant and for these 'late' cases there was no suggestion of a difference in vitamin E levels between subjects and controls for cancer at any of the specified sites (Table I). Indeed, the subject-control differences in these 'late' cases and the 'early' ones diagnosed before the elapse of one year since blood collection were statistically significantly different ($P=0.01$) suggesting a real difference in effect between the two groups.

**Table I** Mean serum vitamin E concentration ($mg l^{-1}$) in cancer subjects and matched controls according to interval between blood collection and diagnosis of cancer and according to site of cancer

| Site of cancer | | Before 1 year: No. men | Before 1 year: Mean vit E | Before 1 year: Diff[a] | 1–2 years: No. men | 1–2 years: Mean vit E | 1–2 years: Diff[a] | 3 or more years: No. men | 3 or more years: Mean vit E | 3 or more years: Diff[a] | All periods: No. men | All periods: Mean vit E | All periods: Diff[a] |
|---|---|---|---|---|---|---|---|---|---|---|---|---|---|
| Lung | Subjects | 9 | 9.2 | −1.5 | 12 | 9.3 | −0.8 | 29 | 9.0 | +0.1 | 50 | 9.1 | −0.4 |
| | Controls | 17 | 10.7 | | 24 | 10.1 | | 58 | 8.9 | | 99 | 9.5 | |
| Colo-rectal | Subjects | 6 | 10.7 | −1.5 | 8 | 11.6 | +1.9 | 16 | 9.5 | −1.1 | 30 | 10.3 | −0.4 |
| | Controls | 12 | 12.2 | | 15 | 9.7 | | 32 | 10.6 | | 59 | 10.7 | |
| Stomach | Subjects | 3 | 11.3 | −0.8 | 5 | 13.5 | +2.5 | 5 | 9.4 | −0.7 | 13 | 11.4 | +0.5 |
| | Controls | 6 | 12.1 | | 10 | 11.0 | | 10 | 10.1 | | 26 | 10.9 | |
| Bladder | Subjects | 8 | 10.7 | +0.3 | 3 | 9.4 | −2.5 | 4 | 11.4 | +2.0 | 15 | 10.6 | +0.2 |
| | Controls | 15 | 10.4 | | 6 | 11.9 | | 8 | 9.4 | | 29 | 10.4 | |
| CNS[b] | Subjects | 5 | 8.4 | −2.0 | 3 | 11.3 | +1.1 | 9 | 9.9 | +0.8 | 17 | 9.7 | 0.0 |
| | Controls | 10 | 10.4 | | 6 | 10.2 | | 18 | 9.1 | | 34 | 9.7 | |
| Skin | Subjects | 31 | 10.4 | −1.6 | 9 | 11.1 | −0.4 | 16 | 10.4 | +0.9 | 56 | 10.5 | −0.7 |
| | Controls | 57 | 12.0 | | 18 | 11.5 | | 32 | 9.5 | | 107 | 11.2 | |
| Other sites | Subjects | 28 | 9.8 | −1.7 | 21 | 10.7 | +0.1 | 41 | 9.8 | +0.7 | 90 | 10.0 | −0.2 |
| | Controls | 55 | 11.5 | | 42 | 10.6 | | 82 | 9.1 | | 179 | 10.2 | |
| All sites | Subjects | 90 | 10.0 | −1.5[c] | 61 | 10.8 | +0.2 | 120 | 9.7 | +0.3 | 271 | 10.1 | −0.2 |
| | Controls | 172 | 11.5 | | 121 | 10.6 | | 240 | 9.4 | | 533 | 10.3 | |

Column groups: Diagnosis of cancer from time of blood collection.

[a]Diff = difference; mean in cancer subjects minus mean in controls; [b]Central nervous system; [c]$P = 0.003$ (the only statistically significant difference amongst differences in the marginal totals of the table – each cancer site or each period to diagnosis).

**Table II** Relative risks of cancer according to vitamin E concentration ($mg l^{-1}$) and interval between blood collection and diagnosis of cancer

| Vitamin E concentration: Quintile | Limits ($mg l^{-1}$) | Before one year: No. of subjects | Before one year: No. of controls | Before one year: Relative risk[a] | After one or more years: No. of subjects | After one or more years: No. of controls | After one or more years: Relative risk[a] |
|---|---|---|---|---|---|---|---|
| 1st | <0.5– | 16 | 17 | 1.89 | 42 | 86 | 0.98 |
| 2nd | 7.4– | 18 | 22 | 1.56 | 40 | 82 | 0.98 |
| 3rd | 9.2– | 19 | 37 | 0.95 | 37 | 65 | 1.14 |
| 4th | 10.7– | 20 | 41 | 0.86 | 30 | 70 | 0.84 |
| 5th | 12.5–34.5 | 17 | 55 | 0.52 | 32 | 58 | 1.12 |
| All | <0.5–34.5 | 90 | 172 | 1.00[a] | 181 | 361 | 1.00[a] |

Column groups: Diagnosis of cancer from time of blood collection.

[a]Relative risks take into account the matched design of the study and are expressed relative to the risk in the 'all' category.

The trend in relative risks for cancer subjects diagnosed before 1 year is statistically significant ($P = 0.003$).

Some have suggested that vitamin E expressed as a ratio to the serum cholesterol level (vitamin E is transported in blood mainly by low density lipoprotein) may be biologically more relevant than vitamin E concentration alone (Horwitt *et al.*, 1972). Expressing the results in this way, or adjusting vitamin E levels for serum cholesterol in an analysis of variance, decreased the significance of the difference in vitamin E relating to the 'early' cases but did not alter the conclusions.

Table II shows the number of subjects and controls and relative risk of cancer according to the quintile of serum vitamin E concentration. There was a statistically significant inverse trend in relative risk among the subjects in whom the diagnosis was made before the elapse of one year from blood collection, but this was not the case for those diagnosed later.

### Discussion

We have demonstrated an inverse association between serum vitamin E and the risk of cancer that was restricted to men who were diagnosed as having cancer before the elapse of one year from the date of blood collection. This suggests that the low serum vitamin E levels were a metabolic consequence, rather than a precursor, of the cancer, even

**Table III** Mean vitamin E concentrations ($mg l^{-1}$) in cancer subjects and controls according to age at blood collection

| Age (years) | Cancer subjects: No. men | Cancer subjects: Mean vitamin E | Controls: No. men | Controls: Mean vitamin E | All: No. men | All: Mean vitamin E | All: s.e. |
|---|---|---|---|---|---|---|---|
| 35–59 | 10 | 9.0 | 23 | 10.5 | 33 | 10.0 | 0.5 |
| 40–44 | 27 | 10.3 | 64 | 10.1 | 91 | 10.1 | 0.4 |
| 45–49 | 49 | 10.5 | 83 | 10.2 | 132 | 10.3 | 0.4 |
| 50–54 | 57 | 10.0 | 121 | 10.8 | 178 | 10.5 | 0.3 |
| 55–59 | 63 | 9.7 | 140 | 10.2 | 203 | 10.0 | 0.2 |
| 60–64 | 65 | 10.2 | 102 | 10.2 | 167 | 10.2 | 0.3 |
| All | 271 | 10.1 | 533 | 10.3 | 804 | 10.2 | 0.1 |

**Table IV** Mean vitamin E concentrations ($mg\,l^{-1}$) in cancer subjects and controls according to smoking status and stated cigarette consumption at the time of blood collection

| | *Cancer subjects* | | *Controls* | | *All* | | |
|---|---|---|---|---|---|---|---|
| *Smoking category* | *No. men* | *Mean vitamin E* | *No. men* | *Mean vitamin E* | *No. men* | *Mean vitamin E* | *s.e.* |
| Life-long non-smokers | 47 | 9.9 | 93 | 10.6 | 140 | 10.4 | 0.4 |
| Ex-smokers | 88 | 10.4 | 175 | 10.7 | 263 | 10.6 | 0.3 |
| Smokers of cigarettes alone: | | | | | | | |
| 1–9/day | 14 | 11.4 | 19 | 9.4 | 33 | 10.3 | 0.7 |
| 10–19/day | 20 | 9.1 | 33 | 10.1 | 53 | 9.7 | 0.4 |
| 20–29/day | 19 | 10.0 | 49 | 9.4 | 68 | 9.6 | 0.4 |
| 30 or more/day | 25 | 9.5 | 43 | 9.6 | 68 | 9.6 | 0.3 |
| All | 78 | 9.9 | 144 | 9.6 | 222 | 9.7 | 0.2 |
| Other smokers | 58 | 9.9 | 121 | 10.4 | 179 | 10.3 | 0.3 |

**Table V** Mean vitamin E concentrations ($mg\,l^{-1}$) in cancer subjects and controls according to duration of storage of the serum sample

| *Storage time (years)* | *Cancer subjects No. men* | *Cancer subjects Mean vitamin E* | *Controls No. men* | *Controls Mean vitamin E* | *All No. men* | *All Mean vitamin E* | *s.e.* |
|---|---|---|---|---|---|---|---|
| <3 | 26 | 10.1 | 50 | 11.0 | 76 | 10.7 | 0.3 |
| 3– | 24 | 12.6 | 50 | 12.2 | 74 | 12.3 | 0.6 |
| 4– | 37 | 10.2 | 66 | 11.4 | 103 | 11.0 | 0.4 |
| 5– | 60 | 10.7 | 122 | 11.3 | 182 | 11.1 | 0.3 |
| 6– | 38 | 8.9 | 72 | 8.8 | 110 | 8.8 | 0.3 |
| 7– | 32 | 9.8 | 66 | 10.5 | 98 | 10.3 | 0.4 |
| 8– | 30 | 9.7 | 58 | 8.5 | 88 | 8.9 | 0.4 |
| ≥9 | 24 | 8.1 | 49 | 8.0 | 73 | 8.1 | 0.4 |

though the cancer may not have been symptomatic or clinically apparent when the blood sample was collected. This conclusion is supported by the fact that vitamin E levels were similarly low in the 50 clinically prevalent cases (including 23 skin cancers) at the time of blood collection and in the 40 cancers that were diagnosed afterwards but still within one year (including 8 skin cancers).

Our results suggest that it is unlikely that serum vitamin E in the concentrations naturally found in well nourished populations has any substantial effect on the risk of developing cancer. It follows that any cancer inhibitory effect suggested by the anti-oxidant activity of vitamin E or by some of the animal experimental evidence is not apparent at levels naturally found in man.

The results of this study, together with those previously published on serum retinol and cancer (Wald *et al.*, 1980) and those on serum cholesterol and cancer (Rose & Shipley, 1980), demonstrate the importance of considering the relationship between a biochemical measurement in subjects who develop cancer according to the interval between blood collection and diagnosis of the cancer. Only by doing so can cause and effect be distinguished when an association between such a measurement and the incidence of cancer is found.

In the design of our study, we matched subjects with controls for age, smoking habits and duration of storage of the serum sample. Mean vitamin E concentrations according to age at the time of blood collection showed no consistent pattern (or significant differences) (Table III); Table IV shows the mean vitamin E levels according to smoking category. Again, there was no clear pattern, though there was a suggestion that serum vitamin E levels were lower in smokers than in non-smokers. Table V shows the mean vitamin E levels according to duration of storage of the serum sample. There was a general decline in vitamin E concentration with increasing storage time; on average, the concentration declined by $0.47\,mg\,l^{-1}$ (or ~5%) per year. Therefore, matching for duration of storage was critical while matching for age or smoking habits was much less so.

Table VI summarises the prospective epidemiological evidence on serum vitamin E and cancer. Two of the seven studied showed statistically significantly lower serum vitamin E levels in subjects who developed cancer compared with controls who did not. Although the other five studies individually did not show statistically significant differences, four yielded differences in the same direction and one

**Table VI** A summary of the epidemiological studies of serum vitamin E and cancer

| *Study* | *Sex* | *Site of cancer* | *No. of cancer subjects* | *No. of controls* | *Approximate mean time to diagnosis of cancer (years)* | *Mean difference in vitamin E ($mg\,l^{-1}$). Cancer subjects minus controls. (approximate s.e.)* | *Published statistical significance* |
|---|---|---|---|---|---|---|---|
| Stähelin *et al.* (1984) | Male | All | 115 | 308 | 4 | −0.9 (0.5) | NS |
| Wald *et al.* (1984) | Female | Breast | 39 | 78 | 5 | −1.3 (0.5) | $P<0.025$ |
| Willett *et al.* (1984) | Both | All | 111 | 210 | 3 | −1.0 (0.6) | NS |
| Nomura *et al.* (1985) | Male | 5 Sites | 284 | 302 | 5 | 0.0 (0.3)[b] | NS |
| Salonen *et al.* (1985) | Both | All | 51 | 51 | 2 | −0.1 (0.3)[a] | NS |
| Menkes *et al.* (1986) | Both | Lung | 99 | 196 | 5 | −1.4 (0.5) | $P<0.001$ |
| Present study | Male | All | 271 | 533 | 3 | −0.2 (0.3) | NS |
| All | | | | | | −0.43 (0.14)[c] | $P=0.003$ |

[a]Standard error (s.e.) was based on a vitamin E standard deviation of $1.6\,mg\,l^{-1}$ estimated from one published $P$ value; [b]s.e. was based on a vitamin E standard deviation of $4.0\,mg\,l^{-1}$ as found in the present study; [c]The overall average across studies was calculated as an average of the individual mean differences, each weighted inversely according to its variance; NS = not statistically significant ($P>0.05$).

showed no difference at all. Taken as a whole, the seven studies show an inverse association between serum vitamin E and cancer, an association which is unlikely to be due to chance. Our own results suggest one explanation for this association, namely that the cancer caused the low vitamin E levels rather than the reverse. It is, however, probably not the only explanation. It is not, for example, a satisfactory explanation for the inverse association shown between plasma vitamin E and breast cancer in women reported by Wald *et al.*, 1984 (a result which requires independent corroboration) because only 6 of 43 cases were diagnosed within 2 years of blood collection. The extent to which it can offer a full explanation for the results from the other studies cited in Table VI would rest on the outcome of a statistical analysis of the differences in vitamin E levels in cancer subjects and controls in these studies classified by time to diagnosis.

We thank Dr R.M. Salkeld and Dr J.P. Vuilleumier of Hoffmann-La Roche, Basle, Switzerland for performing the vitamin E assays, and Mr P. Thompson for technical assistance. We thank the Imperial Cancer Research Fund and the British United Provident Association for financial support.

### References

BRESLOW, N.E. & DAY, N.E. (1980). *Statistical Methods in Cancer Research*, Vol. 1. p. 247. The Analysis of Case-Control Studies, IARC: Lyon.

BURTON, G.W., CHEESEMAN, K.H., DOBA, T. & INGOLD, U. (1983). Vitamin E as an antioxidant *in vitro* and *in vivo*. In *Biology of Vitamin E*, Porter, R. & Whelan, J. (eds) Ciba Foundation symposium 101, p. 4. Pitman: London.

BURTON, G.W. & INGOLD, K.U. (1981). Autoxidation of biological molecules. 1. The antioxidant activity of vitamin E and related chain-breaking phenolic antioxidants *in vitro*. *J. Am. Chem. Soc.*, **103**, 6472.

COOK, M.G. & MCNAMARA, P. (1980). Effect of dietary vitamin E on dimethylhydrazine-induced colonic tumours in mice. *Cancer Research*, **40**, 1329.

DIPLOCK, A.T. (1983). The role of vitamin E in biological membranes. In *Biology of Vitamin E*, Porter, R. & Whelan, J. (eds) Ciba Foundation symposium 101, p. 45. Pitman: London.

HABER, S.L. & WISSLER, R.W. (1962). Effect of vitamin E on carcinogenicity of methylcholanthrene. *Proc. Soc. Exp. Biol. & Med.*, **111**, 774.

HARMON, D. (1969). Dimethylbenzanthracene-induced cancer: inhibiting effect of dietary vitamin E. *Clin. Res.*, **17**, 125.

HORWITT, M.K., HARVEY, C.C., DAHM, C.H. & SEARCY, M.T. (1972). Relationship between tocopherol and serum lipid levels for determination of nutritional adequacy. *Ann. N. Y. Acad. Sci..*, **203**, 223.

MENKES, M.S., COMSTOCK, G.W., VUILLEUMIER, J.P., HELSING, K.J., RIDER, A. & BROOKMEYER, R. (1986). Serum beta-carotene, vitamins A and E, selenium, and the risk of lung cancer. *New Engl. J. Med.*, **315**, 1250.

NOMURA, A.M.Y., STEMMERMANN, G.N., HEILBRUN, L.K., SALKELD, R.M. & VUILLEUMIER, J.P. (1985). Serum vitamin levels and the risk of cancer of specific sites in men of Japanese ancestry in Hawaii. *Cancer Research*, **45**, 2369.

REDDY, B.S. & TANAKA, T. (1986). Interactions of selenium deficiency, vitamin E, polyunsaturated fat, and saturated fat on azoxymethane-induced colon carcinogenesis in male F344 rats. *J. Nat. Cancer Inst.*, **76**, 1157.

ROSE, G. & SHIPLEY, M.J. (1980). Plasma lipids and mortality: a source of error. *Lancet*, **i**, 523.

SALONEN, J.T., SALONEN, R., LAPPETELAINEN, R., MAENPAA, P.H., ALFTHAN, G. & PUSKA, P. (1985). Risk of cancer in relation to serum concentrations of selenium and vitamins A and E: matched case-control analysis of prospective data. *Br. Med. J.*, **290**, 417.

STÄHELIN, H.B., ROSEL, F., BUESS, E. & BRUBACHER, G. (1984). Cancer, vitamins, and plasma lipids: prospective Basel study. *J. Nat. Cancer Inst.*, **73**, 1463.

TOTH, B. & PATIL, K. (1983). Enhancing effect of vitamin E on murine intestinal tumorigenesis by 1,2-dimethylhydrazine dihydrochloride. *J. Nat. Cancer Inst.*, **70**, 1107.

VIULLEUMIER, J.P., KELLER, H.E., GYSEL, D. & HUNZIKER, F. (1983). Clinical chemical methods for the routine assessment of the vitamin status in human populations. Part I. The fat-soluble vitamins A and E, and beta-carotene. *Int. J. Vit. Nutr. Res.*, **53**, 265.

WALD, N., BOREHAM, J. & BAILEY, A. (1986). Serum retinol and subsequent risk of cancer. *Br. J. Cancer*, **54**, 957.

WALD, N., BOREHAM, J., HAYWARD, J.L. & BULBROOK, R.D. (1984). Plasma retinol, beta-carotene and vitamin E levels in relation to the future risk of breast cancer. *Br. J. Cancer*, **49**, 321.

WALD, N., IDLE, M., BOREHAM, J. & BAILEY, A. (1980). Low serum-vitamin-A and subsequent risk of cancer. Preliminary results of a prospective study. *Lancet*, **ii**, 813.

WILLETT, W.C., POLK, B.F., UNDERWOOD, B.A. & 5 others (1984). Relation of serum vitamins A and E and carotenoids to the risk of cancer. *New Engl. J. Med.*, **310**, 430.